# Factors affecting feeding ability in children with neonatal intensive care unit stay: a cluster analysis using machine learning methods

**DOI:** 10.3389/fped.2025.1578612

**Published:** 2025-11-07

**Authors:** Jinwei Feng, Tian Shu, Ruihao Li, Xuan Feng, Yi Huang, Jianguo Cao, Kanglong Peng

**Affiliations:** 1 Rehabilitation Department, Shenzhen Children's Hospital, Shenzhen, Guangdong, China; 2 Jiangxi Education Evaluation And Assessment Institute, Nanchang, Jiangxi, China

**Keywords:** oral feeding, NICU, neonates, cluster analysis, XGBoost, SHAP value

## Abstract

**Objectives:**

Oral feeding introduction is challenging in Neonates Intensive Care Unit (NICU) daily care with limited measuring methods. Our study aimed to depict the oral feeding related features in neonates with critical conditions who were administered to NICU and its major predictors.

**Study design:**

A total of 1,419 neonates with critical conditions who were administered to NICU were enrolled. The related features were acquired by using the Preterm Infant Oral Feeding readiness assessment scale (PIOFRA). The Shapley Additive Explanation (SHAP) values were used in XGBoost models established based on selected features. In addition, the ANOVA analysis was adopted to depict the group differences.

**Results:**

Three profiles with distinct PIOFRA features were identified in cluster analysis (*p* > 0.05). Compared to other prediction models (e.g., Logistic Regression, Random Forest), the XGBoost model achieved the highest accuracy (85.2%). Sucking power and rooting reflex were identified as the features with largest impact in oral feeding predations that exhibited positive and negative influence respectively.

**Conclusions:**

Oral feeding difficulty can be commonly observed in neonates in NICU, and more detailed assessments are needed to illustrate the difference in gestational features (e.g., born weight, gestational age) between difference profiles. PIOFRA features can be strong predictors in predicting whether neonates had achieved full oral feedings or not. However, more studies are needed to verify the detailed mechanism to illustrate how sucking and rooting reflex functions ensure the safe and efficient content transportation in neonates administered to NICU.

## Introduction

Infants with prolonged neonatal intensive care unit (NICU) stay are more likely to display abnormal developmental trajectory comparing to typical developed peer after discharged from NICU, and more complicated problems may emerge as they grow older (1, 2). Longitudinal findings reveal that developmental trajectory may vary among these population that some may deviate downward from average curve, and some may also try to approach the normal ones as well, but most cases commonly remain the same suboptimal developmental outcomes (3, 4).

Eating is a highly complicated motor skill that involves preparatory phases, oral phases, pharyngeal phases, esophageal phases, and gastric phases. Typically developed infants can achieve independent oral feeding skills by using well-coordinated motor functions. In preterm infants, N Amaizu found that infants with 26–29 weeks gestational age can obtain oral feeding when clinically stable (5). Occasionally, breast- or bottle-feeding in infants should not be a common concern, however one or more unexpected failures in feeding process can result in feeding problems. Hence, over 25%–45% of normal developing children and up to 80% of developmental delayed children can experience feeding problems (6). The timing when oral feeding is introduced mainly depends on the coordination among necessary functions such as sucking, swallowing, respiration, and etc. (5).

Problematic feedings were commonly found in preterm infants (e.g., <37 weeks' gestation) (7). The feeding status among infants born less than 28 weeks was so terrible that over 50% of these infants needed tube feeding after discharging (8). Feeding problems can also be found in term infants with NICU stay (9). Study found that some maternal and neonatal factors may be considered as obstacles to satisfied oral feeds (e.g., breast- or bottle-feeding), for example suboptimal maternal education, delay physical contact, and transient neonatal feeding intolerance can produce negative impacts on early oral feedings (10, 11). The normal feeding process can be jeopardized in a preterm or term infant by extrauterine impact leading to dysphagia with malfunctioning sucking, swallowing, or cough reflexes, thus compromising the efficacy, adequacy, and the safety of oral feedings (12). Hence, achieving competent oral feeding is one of the main determinants that affect discharge from NICU (13). Suboptimal feeding experiences can not only extend the hospital stay but also increase the unnecessary NICU readmission (14).

Feeding problems can produce continuous impacts on the developmental growth of infants, hence necessary assessments and interventions are needed to provide optimal managements (12). For now, the maturational processes in oral feeding remain inconclusive (7, 9). Clinical guidelines for oral feeding vary in practical recommendations and interventions details (15). This can be explained by the fact that only limited studies adopted standardized assessments to present the oral feedings functions in neonates (7). On the other hand, the heterogeneity across these studies may reflect the fact that these standardized assessments (e.g., Schedule for Oral Motor Assessment, Montreal Children's Hospital Feeding Scale) cannot clearly depict the oral feedings difficulties in neonates with critical conditions (7). Another reason is that previous study mostly utilized univariate analysis that cannot identify reliable factors that contribute to classify infants with or without oral feeding ability, besides small sample sizes used in these study also limit the generalization of their findings (16).

How to facilitate early oral feedings in infants with NICU stay remains unsolved for NICU health care professionals. To address the ambiguous definition of oral feeding disorders, our study adopted the consensus definition proposed by Goday and colleagues: impaired oral intakes that is not age-appropriate, and is associated with medical, nutritional, feeding skill and/or psychosocial dysfunction (17). Ayse Ecevit found that swallowing in infants can yield valuable evidence for clinical decisions regarding safe discharge (e.g., obtain oral feeding) using a small sample size (18). To make amendments to previous findings, our study conducted a large epidemiological study using one comprehensive and psychometrically-sound assessment to clearly depict the overall pictures of feedings in neonates with critical conditions.

Our study applied a machine learning classifier, the Extreme Gradient Boosting algorithm (XGBoost) to determine whether an infant administered to NICU can obtain early oral feeding or not. This method was chosen mainly based on its outperforming efficiency compared to other algorithms, and it was widely used in other studies (19–21). To our knowledge, there are no studies using XGBoost to classify oral feeding ability in infants administered to NICU. Our study aimed to provide more insightful evidence to unveil the factors that contribute to oral feedings in infants with NICU stays. Further, our findings may contribute to developing preventive and therapeutic interventions that can facilitate safe transition from tube feedings to oral feedings.

## Methods

### Study design and setting

A cross-section study was conducted in NICU of one local public hospital.

### Participants

Participants were recruited from referral programs in hospital. On the first day of administration, infants admitted in NICU were referral for early rehabilitation through this program and would receive interdisciplinary assessment for overall neurobehaviors status and join the follow-up program to obtain necessary suggestions. The comprehensive assessment routinely involves the administration of early evaluation for readiness for early oral feeding. Prior to administration, all necessary consents were obtained from their legal guardian(s).

### Inclusion and exclusion criteria

This study included infants who were born at 28–39+ gestational age with critical conditions (e.g., infectious or parasitic diseases) and admitted to NICU. Neonates who died in their immediate postnatal day were excluded.

### Measure

The Preterm Infant Oral Feeding readiness assessment scale (PIOFRA or PRIOFRAS) was built for serving as an observation rating tool to quantify the oral feeding readiness in infants admitted to NICU (22). The Chinese PIOFRA was built in 2013 with acceptable psychometric properties (Cronbach's alpha: 0.804) (23–25). This tool is applicable to help clinicians to determine whether infants can achieve independent oral feedings (e.g., breast or bottle) (26). PIOFAR consists of the following components: gestational age, behavior status, oral function, oral reflex, and sucking. The total score is a sum of all items where scores under 30 (e.g., cut-off values: 29, sensitivity: 0.938, specificity: 0.941) denote oral feeding intolerance (23, 24, 27). This test was conducted when infant is awake to ensure trustful response to PIOFRA.

### Data analysis

#### Cluster analysis

Our study utilized the Ward Linkage method or the minimum variance method to detect the potential cluster in our sample. To determine the optimal cluster amount, we used the classical elbow method based on Bayesian and Akaike information criteria. The ANOVA analysis was conducted to depict the parameters differences among clusters, and significant level was set at *p*-values lower than 0.05 in all tests. The Bonferroni *post hoc* analysis was conducted to depict the difference between clusters.

#### Gradient-boosting machines learning method for prediction

The machine learning methods are used to establish a formula to simulate interactions among selected features and outcomes. Our study tried to simulate the relations between related factors and oral feeding outcome, or we tried to determine whether an infant administered to NICU is suitable for early oral feedings or not based on involved factors. More precisely, we tried to obtain the most optimal combination of selected features that demonstrate the most powerful prediction in binary classification. The XGBoost is an ensemble learning algorithm based on gradient-boosting decision tree using continuous repetition process to obtain the least prediction error to achieve higher prediction accuracy.

The feeding intolerance prediction model was built based on the XGBoost algorithm. The Shapley Additive Explanation values were extracted regarding the impact of involved parameters in predictions (e.g., magnitude, direction).

## Result

### Sample characteristics

Table 1 displays the sample characteristics in our study. This study collected a sample mainly consisting of boys (*N* = 868, 61.2%). Overall, we managed to collect 1,052 neonates with abnormal birth weight (*N* = 1,052, 74.1%). The average gestational age is around 38 weeks, that means we have only collected termed infants (e.g., around 39 weeks). The average chronological age is around 38 days old, that means infants were administered to NICU in the first months of their lives.

**Table 1:** Participant demographic data.

Variables	Total	Clusters			Oral	Non-Oral
		Mild	Moderate	Severe		
Sample	1,419	793	578	48	429	990
Gestational age	219.68 (38.98)	218.47 (38.35)	220.17 (39.86)	233.67 (36.5)	225.9 (39.7)	217 (38.4)
Chronological age	47.85 (45.57)	45.67 (44.43)	50.79 (47.9)	48.6 (32.09)	47 (49.2)	48.2 (43.9)
Gender						
Female	551	318 (40.1)	208 (36)	25 (52.1)	168	383
Male	868	475 (59.9)	370 (64)	23 (47.9)	261	607
Born weight	2,109.7 (854.4)	2,109.32 (878.17)	2,093.92 (834.2)	2,305.94 (665.86)	2,260.5 (840.6)	2,044.4 (852.5)
Normal birth weight	367 (25.86)	205 (25.9)	150 (26)	12 (25)	126 (29.4)	241 (24.3)
Low birth weight (<2,500)	616 (43.41)	342 (43.1)	245 (42.4)	29 (60.4)	201 (46.9)	415 (41.9)
Very low birth weight (<1,500)	250 (17.62)	139 (17.5)	109 (18.9)	2 (4.2)	60 (14)	190 (19.2)
Extremely low birthweight (<1,000)	186 (13.11)	107 (13.5)	74 (12.8)	5 (10.4)	42 (9.8)	144 (14.5)
ICD_Diagnosis						
Certain conditions originating in the perinatal period	804 (56.7)	483 (60.9)	306 (52.9)	15 (31.3)	250 (58.3)	554 (56)
Certain infectious or parasitic diseases	26 (1.8)	12 (1.5)	13 (2.2)	1 (2.1)	11 (2.6)	15 (1.5)
Developmental anomalies	128 (9)	60 (7.6)	64 (11.1)	4 (8.3)	18 (4.2)	110 (11.1)
Diseases of the blood or blood forming organs	2 (0.1)	1 (0.1)	1 (0.2)	–	1 (0.2)	1 (0.1)
Diseases of the circulatory system	9 (0.6)	4 (0.5)	5 (0.9)	–	4 (0.9)	5 (0.5)
Diseases of the digestive system	8 (0.6)	3 (0.4)	4 (0.7)	1 (2.1)	2 (0.5)	6 (0.6)
Diseases of the genitourinary system	2 (0.1)	1 (0.1)	1 (0.2)	–	0	2 (0.2)
Diseases of the nervous system	70 (5)	33 (4.2)	27 (4.7)	11 (22.9)	27 (6.3)	44 (4.4)
Diseases of the respiratory system	119 (8.4)	55 (6.9)	53 (9.2)	11 (22.9)	37 (8.6)	82 (8.3)
Diseases of the visual system	17 (1.2)	9 (1.1)	8 (1.4)	–	13 (3)	4 (0.4)
Endocrine, nutritional or metabolic diseases	17 (1.2)	11 (1.4)	6 (1)	–	10 (2.3)	7 (0.7)
Symptoms, signs or clinical findings, not elsewhere	216 (15.2)	121 (15.3)	90 (15.6)	5 (10.4)	56 (13.1)	160 (16.2)
Feeding status						
Non-oral feeding	990 (69.8)	516 (65.1)	432 (74.7)	42 (87.5)	–	–
Oral feeding	429 (30.2)	277 (34.9)	146 (25.3)	6 (12.5)	–	–

Over 804 neonates were administrated to NICU due to certain conditions originating in the perinatal period (e.g., disorders of newborn related to length of gestation). In these neonates, most of them (*N* = 990, 69.8%) cannot achieve oral feeding (e.g., breast or bottle). No neonates who died in their immediate postnatal day in our study. In this sample, only 429 neonates (30.2%) who are aged around 47-days old can achieve oral feeding (e.g., breast or bottle).

**Figure 1:**
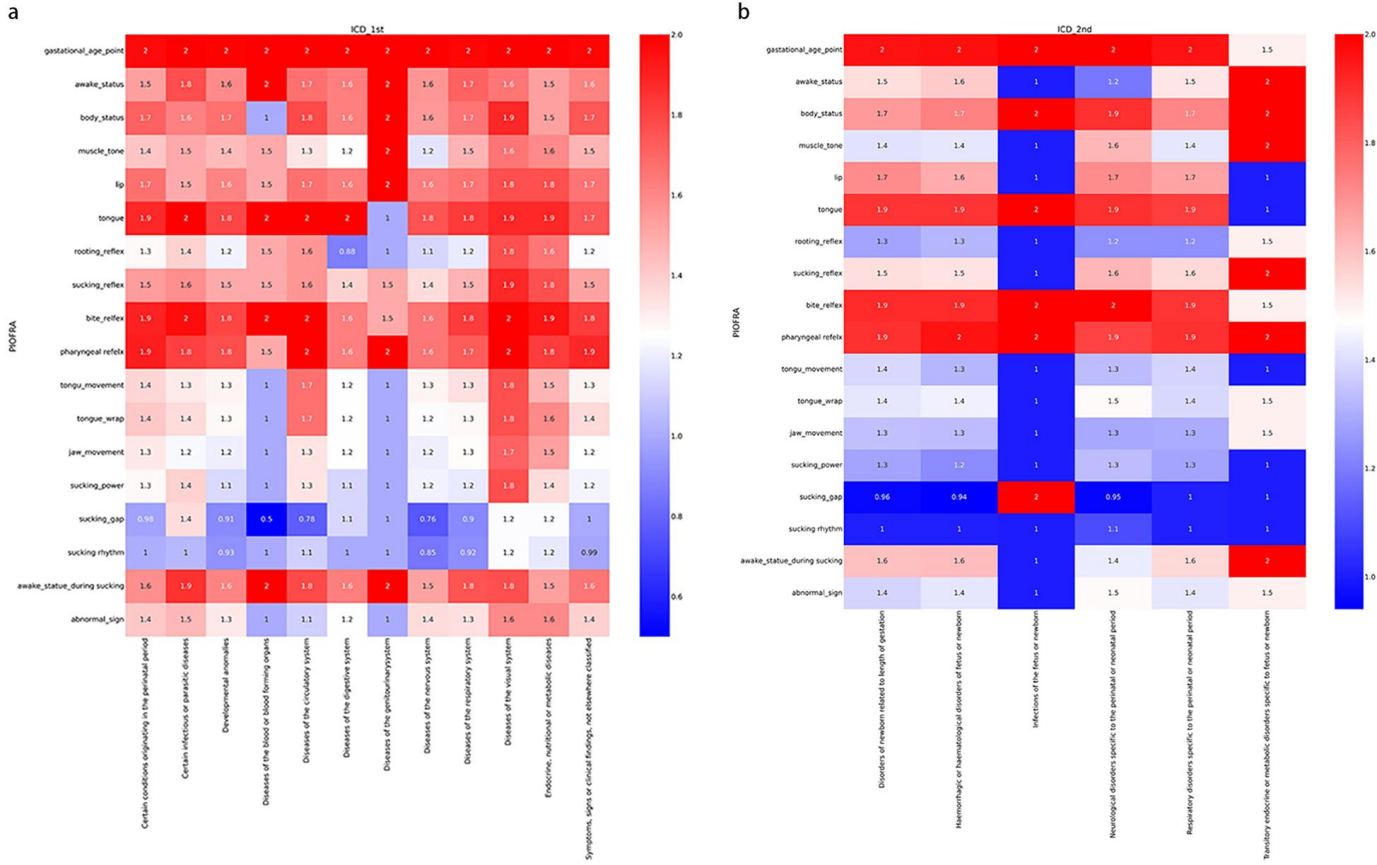
Performance of the collected participants in PIOFRA in different subsample categorized according to ICD 11th. **(a)** International Classification of Diseases 1st level. **(b)** International Classification of Diseases 2nd level under certain conditions originating in the perinatal period.

Figure 1 depicts the overall performance of the collected participants in PIOFRA according to International Classification of Diseases 11th version (ICD 11th). The colors indicated the average scoring points ranged from 0 to 2, and each row stands for one testing item. Overall, we noticed that similar performance across different diagnosis that neonates in NICU display suboptimal reflex and sucking behaviors. Our study conducted the *t*-test analysis to depict the difference between neonates who can or cannot achieve oral feedings, and our results found that neonates achieved oral feedings were scoring significant high points than those who were fed by gastric tube in PIOFRA total scores. Infants who obtained oral feedings scored 39.58 ± 5.51 points while those who cannot achieved 35.79 ± 6.89 (*p* equals to 0.00).

### Using cluster analysis to identify clusters in samples

**Figure 2:**
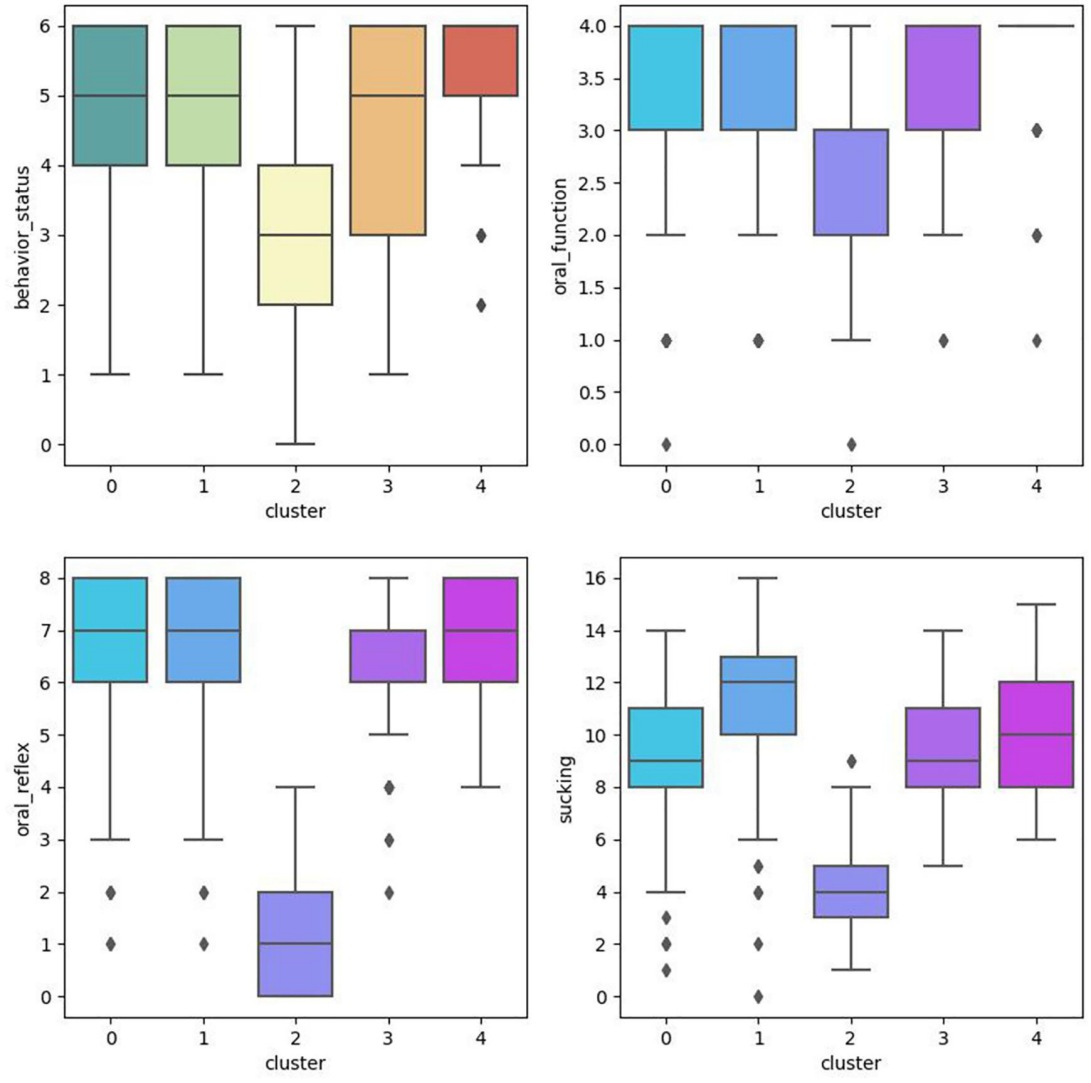
ANOVA analysis among clusters.

This study conducted cluster analysis and ANOVA analysis to identify homogeneous subgroups in this sample based on PIOFRA performance. The elbow method was adopted to obtain the optimal numbers of clusters based on the Arkia information criteria and Bayesian information criteria. Five clusters were detected in this sample. Table 2 describes the performance patterns based on the PIOFRA assessment results (Figure 2).

**Table 2:** Cluster characteristics based on PIOFRA performance and demographic data.

Cluster	Profile	Total (*n*, %)	Oral_feeding (*n*)	Gender/Male	Age (day)	Gestational_age (day)	Born weight (g)	Measure parameter				
								Behavior_status	Oral_function	Oral_reflex	Sucking	Total
0	Moderate	433	110	269	50.8 (48.6)	222.3 (40.4)	2,149 (835.7)	4.68 (0.06)	3.44 (0.04)	6.39 (0.07)	9.46 (0.11)	25.94 (0.2)
1	Mild	680	240	402	45.2 (44.9)	219.6 (38.6)	2,134.8 (880.2)	4.82 (0.04)	3.55 (0.03)	6.95 (0.05)	11.39 (0.09)	28.7 (0.16)
2	Severe	48	6	23	48.6 (32.1)	233.7 (36.5)	2,305.9 (665.9)	2.88 (0.22)	2.83 (0.14)	1.27 (0.19)	4 (0.27)	12.98 (0.54)
3	Moderate	145	36	101	50.9 (45.8)	213.9 (37.7)	1,929.3 (810.3)	4.44 (0.12)	3.49 (0.05)	6.22 (0.1)	9.37 (0.18)	25.48 (0.33)
4	Mild	113	37	73	48.2 (41.5)	211.9 (36.2)	1,956.2 (853.4)	5.19 (0.09)	3.7 6 (0.05)	6.88 (0.09)	10.02 (0.19)	27.8 (0.32)
		F			1.17	3.97	3.56	35.21	14.98	230.62	145.91	186.91
		p			0.32	0	0.01	0.000	0.000	0.000	0.000	0.000
		Bonferroni post-hot			–	2 > 3, 4	–	4 > 1>3 > 2 4 > 0>2	4 > 3, 0 > 2 1 > 2	1, 4 > 0, 3 > 2	1, 4 > 0, 3 > 2	1, 4 > 0, 3>

Cluster 1 and 4 were scoring the highest points in PIOFRA, and cluster 2 performed the worst compared to other clusters. In the mild groups, cluster 1 stood for participants displays the worse performance in behavior status, and cluster 4 represented those with suboptimal sucking behaviors. No significant difference was found among these clusters in born weight and age. Only the severe group displayed the longest gestational days compared to cluster 3 and 4.

### Using XGBoost methods to establish models for oral feeding prediction

**Figure 3:**
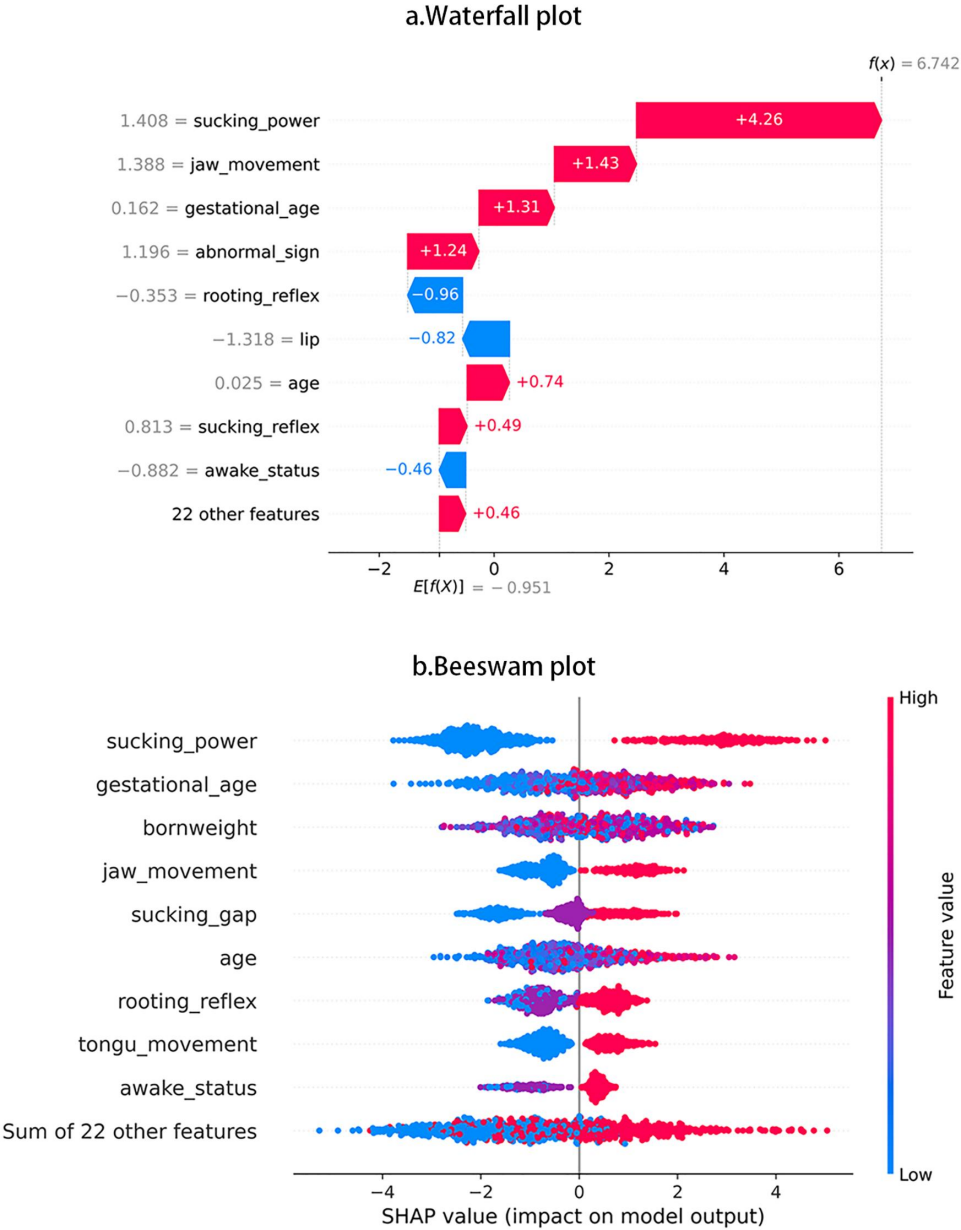
Interpretation of SHAP value plot in prediction. **(a)** Waterfall plot, **(b)** Beeswam plot.

One XGBoost model was established to predict the following binary categories: oral feedings or non-oral feedings. The ultimate accuracy score was 85.2%. Figure 3a displays the features’ impact on oral feeding predictions in XGBoost. These features were ranked in descending order according to the impact magnitude. Red-colored bars indicated positive impact, and the blue ones presented negative influences. The bar width of the colored regions stood for the impact magnitudes, and each dot in Figure 3b stood for one sample.

To visually illustrate the feature impacts, this study would provide some typical examples. Figure 1a shows that sucking power contributed the largest impact on prediction accuracy, then Figure 1b shows that, as sucking power increases, SHAP values show increasing trend and indicate higher possibility of achieving oral feedings. Figure 1a also shows that rooting reflex produced the largest negative impact, and Figure 1b shows that some participants with more optimal rooting reflex tend to display worse oral feeding performance.

## Discussion

Our study aimed to depict the possible related features in oral feedings in neonates with NICU stay. Our study utilized PIOFRA to obtain those important features for the following analysis. At first, the cluster analysis based on Ward's method was used to find the distinct cluster traits. Secondly, the ANOVA analysis is used to reveal the difference among clusters. Lastly, our study established one prediction model based on XGBoost method and used SHAP values to depict the features' impacts on predictions accuracy. This study identified three distinct performance profiles, i.e., mild, moderate, and severe in a sample of neonates with critical conditions in NICU. Mild profiles denoted neonates with the least impaired oral feeding functions, and some may display unpleasant behavior status or suboptimal sucking movements. Moderate profiles display medium PIOFRA performance. Individuals with mild or moderate profiles may display the same frequent abnormal signs, which are not occasionally found in those with severe profiles. Our findings also highlight the importance of sucking power in oral feeding.

### The PIOFAR features in neonates with critical conditions in NICU

PIOFRA is considered as the first assessment that provide clinical details to illustrate the oral feedings in neonates including term and preterm infants and decide when to start breast or bottle feeding (22, 28). PIOFAR contains the following components: gestational age, behavior status, oral function, oral reflex, and sucking.

Previous findings pointed out that most neonates including term and preterm infants can achieve oral feedings by term-equivalent age (29). In addition to previous findings, our study pointed out that neonates with suboptimal birth weight or insufficient gestational period may be less likely to achieve oral feedings at term-equivalent age compared to those with more satisfied conditions.

For behavior status, studies found that comforting body position may affect the gastric emptying rate or the amount of gastric residue (30). That is reason why we found that some infants with enteral nutrition may display abnormal positions (e.g., extremities extension, hyperkinetic or hypokinetic reactions). Our findings confirmed that comforting body positions can produce consideration impact on satisfying oral feeding by facilitating biological gastric movements. For the rest subscales, prior studies have noticed the importance of oral functions, reflexes, and sucking in oral feedings (31, 32). The findings in this study reflect that oral dysfunction is highly prevalent in the neonates regardless of various critical conditions. In brief, our results confirm that problematic oral feeding is highly common in neonates with critical conditions, especially when they are administered in NICU. One limitation in our study is that the oral feedings features are only captured by one standardized assessment, and that is reason why homogeneity is found across individuals with different conditions.

### PIOFRA performance in individuals with different symptom profiles

Using unsupervised learning-based cluster analysis has provided us with new insights into profiles of clinical impairment of oral feedings in neonates with critical conditions in NICU. Clustering based on PIOFRA scoring displays distinct differences in clusters. These observations have not previously been illustrated in neonates administered in NICU. Overall, those who perform worse on one subscale tend to be worse in others. In comparison, we identify some mixed characteristics in mild profile that display different problems in behavior status and sucking. One unexpected findings is that one cluster with shorten gestational age (211.9 ± 36.2 days) but display milder feeding impairment. In line with previous findings, gestational age and born weight can be strong predictors for feeding difficulties, but we still cannot understand how gestational age and born weight are affecting the timing when a suboptimal born weight neonate achieve full oral feedings (33, 34). That is the reason why overall difference is found among profiles, but we cannot find significant differences between different clusters. One unexpected finding is that neonates present the worst PIOFRA performances but process the longest gestational days compared to some cluster in mild and moderate profiles. In summary, using PIOFRA may be applicable in distinguishing phenotypes of oral feedings difficulty in neonates administered to NICU.

### Using PIOFRA features to predict oral feeding in neonates with critical conditions

Previous studies point out that predicting the timing when neonates with critical conditions achieve full oral feedings can be very difficult, some related features (e.g., maternal factors, born weight, infection) are identified as factors that associated with the timing of full oral feeding (33, 35). As previous studies proposed, oral feeding is administered in a way using pragmatic feeding milestone as guiding criteria (36, 37).

In this study, compared with classical statistical model (e.g., regression), the unsupervised machine learning method can achieve higher accuracy to predict whether neonates can achieve oral feedings or not based on cross-sectional information. To promote model interpretability, we utilized the SHAP value to quantify the features impact in XGBoost Model and depict the contribution of these features (e.g., positive or negative).

In this article, we used the PIOFRA features to build the XGBoost prediction model for oral feeding and obtain more satisfying accuracy compared to other machine learning models (e.g., Regression, SVM, Random Forest). Among these PIOFRA features, sucking power is identified as the factor that produces the largest positive impact in predicting oral feeding outcome. This result is also confirmed by previous studies that well-functioning sucking is one of the most vital stages that can ensure the safe and efficient transport of nutrient content (9, 38, 39). In contrast to previous findings, our study reveals that rooting reflex is not always contributing positively to feeding orally (22, 32, 40). This can be partially explained by the fact that rooting reflexes may function mostly as hunger cues in daily feeding that mainly elicited by feeding deprivation and satiation (40). Hence, rooting reflexes may be more obvious in infants who have experienced feeding deprivation, but feeding deprivation would be less likely happened in NICU feeding (e.g., preset volume feeding in scheduled intervals).

### Study limitations

Several limitations are worth mentioning. First, our study adopted the PIOFRA to depict possibly all the related features in oral feeding activities, but our results indicated that the assessment outcome may not be comprehensive enough to produce heterogenous information regarding feedings in infants with critical conditions. Second, the optimal performances in PIOFRA are difficult to fully elicited due to the inherit unstable nature in neonates. In this study, we try to collect a large enough sample to eliminate the potential cofounding factors (e.g., incorrect assessment timing, post-invasive operations). Thirdly, other potential variables related to feeding (e.g., heart rate, blood pressure, oxygen) were missing due to methodological limitations. Besides, due to the cross-sectional nature of this study, other prospective studies are needed to validate our findings. In addition, due to limited assessment methods, it remains unclear that how these related features (e.g., gestational age, born weight, oral traits) contribute to deciding the timing when neonates can achieve fully oral feedings.

### Implication

Our result provided necessary evidence for early support of oral feeding skills in infants administered to NICU. The PIOFRA outcomes can serve as a useful tool to identify subsamples with different oral feeding disorders. To our knowledge, how different modalities of oral feeding difficulties cluster at individual level in a large enough sample has never been fully illustrated before. For example, we have identified two subsamples within mild profiles that perform differently in behavior status and sucking. We also have found the relation between impairment level and other related features (e.g., born weight, gestational age) at profile level. Studies have revealed that early assessment and supportive intervention can promote the start of early oral feeding in infants and the early discharge (41, 42). Hence, illustrating the interactions among feeding performance and related factors during neonatal period allows for a better understanding of potential contributors to early feedings and identifies targets or individualized interventions.

## Conclusion

In summary, Oral feeding difficulty can be commonly observed in neonates in NICU. In this study, we used clustering analysis to distinguish potential groupings with similar measuring features within a relatively large sample. Our results have identified three profiles with distinct PIOFRA characteristics. More detailed assessments are needed to illustrate the difference in gestational features (e.g., born weight, gestational age) between different profiles. As previous studies suggested, oral feedings are commonly provided in a manner using pragmatic feeding milestones as guiding criteria or simply based on cue (e.g., rooting reflex or crying). Hence, it is urgently needed to achieve more reliable evidence to decide the timing when neonates with critical conditions can obtain full oral feedings. In addition, we also have recognized sucking power and rooting reflex as the factor with the largest impact on prediction outcomes, these two features contribute to the model prediction in different directions. More studies are needed to verify the detailed mechanism to illustrate how sucking and rooting reflex functions to ensure the safe and efficient content transportation in neonates administered to NICU.

## Data Availability

The original contributions presented in the study are included in the article/Supplementary Material, further inquiries can be directed to the corresponding author.
